# Major changes in gene expression between strains of diapausing and non-diapause spruce budworm

**DOI:** 10.1371/journal.pone.0349357

**Published:** 2026-08-18

**Authors:** Karin R. L. van der Burg, Jeff Gauthier, Amanda D. Roe, Michel Cusson, Brian Boyle, Roger C. Levesque, Katie E. Marshall

**Affiliations:** 1 Department of Zoology, University of British Columbia, Vancouver, Canada; 2 Department of Biological Sciences, Clemson University, Clemson, United States of America; 3 Institut de Biologie Intégrative et des Systèmes, Université Laval, Québec Québec, Canada; 4 Département de Microbiologie, d’Infectiologie et d’Immunologie, Faculté de Médecine, Université Laval, Quebec, Canada; 5 Natural Resources Canada, Canadian Forest Service, Great Lakes Forestry Centre, Sault Ste. Marie, Ontario, Canada; 6 Natural Resources Canada, Canadian Forest Service, Laurentian Forestry Centre, Quebec City, Quebec, Canada; Laboratoire de Biologie du Développement de Villefranche-sur-Mer, FRANCE

## Abstract

To survive harsh winter conditions, many insects enter a state of dormancy called diapause which allows them to withstand extreme temperatures and lack of food. When diapause is induced, insects enter a state of arrested development with a low metabolic rate. Because diapause and environmental conditions are closely linked, variation within and between species in diapause induction, depth, and duration is extremely common. Studies investigating the genetic underpinnings of diapause tend to focus on either different populations and/or environmental variation. We use the eastern spruce budworm (*Choristoneura fumiferana*) to investigate within-population variation in diapause induction. Typically, *C. fumiferana* diapauses as second instar larvae, but a small subset of individuals do not diapause and instead continues through development. This non-diapause phenotype can be selected to create a non-diapause strain. Here, we present a chromosome-level assembly of such a non-diapause strain and compare it to an earlier published genome assembly of the diapause strain. Despite the very large life history difference, we did not find evidence of major chromosome rearrangements, indicating that the genetic variation between strains is likely small. Gene expression comparisons between the strains indicate major gene expression changes, where genes associated with glycolysis and environmental signaling processing increase in expression in the diapause strain. Lastly, we found that gene expression diverges halfway through the first instar.

## Introduction

Insects living in temperate climates often face stressful seasonal conditions where extreme temperatures and lack of food are common. One adaptation to winter conditions is diapause, a hormone-induced dormant state associated with extensive physiological changes such as arrested development and low metabolic activity [1]. When diapause is induced, insects shift away from a direct developmental program and start to prepare for diapause through changes in gene expression, neuroendocrine signaling, and metabolism until diapause is initiated [2,3]. Most diapausing insects have a facultative diapause program, where an external signal such as photoperiod or temperature during a sensitive period is translated to an internal signal to induce the diapause program [4], although species with an obligate diapause, which is induced regardless of external conditions, do exist [3]. Because diapause and environmental conditions are so closely linked, there is often extensive local adaptation in diapause regulation [5,6]. Thus, studies investigating genetic mechanisms underlying diapause tend to focus on either between-population variation [7,8], where the diapause phenotype is locally adapted, or within-population comparisons where environmental conditions vary to induce or prevent diapause (facultative diapause) [9]. Here, we aim to add to this work by investigating within-population variation of an obligate diapause. This allows us to investigate diapause induction in specimens from the same genetic background, without environmental variation.

Diapause is a complex trait, and significant research has been conducted to resolve its underlying regulation and genetic architecture [10,11]. Generally, diapause consists of three phases: pre-diapause, diapause and diapause [3]. Extensive research has been done on all three stages, but in particular the pre-diapause stage has been well-investigated. Either an external (facultative diapause) or an internal (obligate diapause) signal induced the pre-diapause stage, where direct development is still ongoing but diapause preparation processes are also underway. When preparation is complete, diapause is initiated, and insects enter a state of developmental arrest.

Several studies have implicated circadian clock genes in variation in diapause induction along a latitudinal gradient. Examples include the parasitic wasp *Nasonia vitripennis* [12], the speckled wood butterfly *Pararge aegeria* [5]*,* the flesh fly *Sarcophaga bullata* [13] and several other species [14,15]. The identification of circadian clock genes associated with clinal changes in diapause induction is perhaps unsurprising, as photoperiodism is the most reliable environmental cue to predict seasonal changes and varies significantly with latitude. A few classic studies in *Drosophila melanogaster* identified different signaling genes underpinning diapause induction: insulin-regulated *Dp110* [16], as well as *couch potato*, a gene believed to be involved with ecdysteroid signaling [17]. Although the link with environmental conditions is less clear-cut, both insulin and ecdysteroids are known to be environmentally responsive endocrine signals. However, a lot remains unknown about the molecular processes underlying diapause induction and preparation, and also the genetic changes necessary to switch between obligate and facultative diapause, and diapause free development altogether.

**Fig 1:**
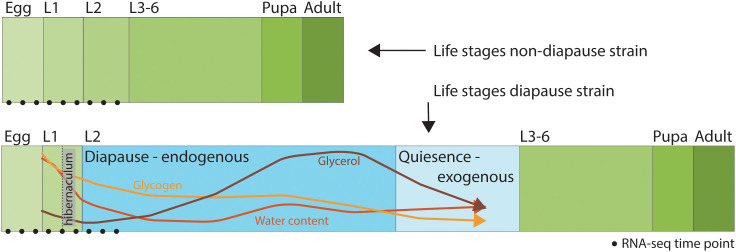
Life stages for the two strains of spruce budworm. Spruce budworm experience periods of active development (green) and developmental arrest (blue). The period of developmental arrest in the diapause strain is an obligatory diapause that occurs during the 2nd larval instar. During diapause relative changes in glycerol, glycogen and water content are shown [18].

The eastern spruce budworm, *Choristoneura fumiferana* (Clemens, 1865, Lepidoptera: Tortricidae), is a widespread conifer pest that enters what is usually considered an obligatory diapause to survive winter throughout its range. Diapause is induced in early instar larvae during late summer and early fall in anticipation of stressful winter conditions, regardless of external conditions. The process of diapause induction and preparation is complex and temperature-sensitive [18–20]. Typically, C. fumiferana is described as univoltine, with one single obligatory diapause [19]. After hatching, larvae forego feeding and seek an overwintering site on the host plant where they construct a silken structure (hibernaculum) prior to molting and spinning a second hibernaculum layer before becoming dormant [21]. However, variation in diapause expression exists within this species (Fig 1). At one extreme, additional diapause stages may occur following emergence in spring, requiring two years for an individual to complete development [22]. At the other end of this continuum, diapause free development (non-diapause) was documented in multiple laboratory rearings of *C. fumiferana* [23]. Here, second instar larvae were able to continue development and did not require a period of developmental arrest. This phenotype could be increased in frequency through exposing larvae to continuous light. Interest in this non-diapause phenotype led Harvey to develop a non-diapause colony of *C. fumiferana*. He used continuous light to select for non-diapause development from *C. fumiferana* families that showed elevated expression of this phenotype. After 12 generations of selection, this colony showed nearly complete non-diapause expression. Because the selection experiment showed such rapid change, it can be assumed that artificial selection acted on standing genetic variation within this population, rather than novel mutations [23]. Within population variation in phenotypes is a valuable tool for in-depth investigations of genetic mechanisms underlying such traits, as there are no confounding factors due to isolation-by-distance or environmental effects [24,25]. In this case, we have within population variation of diapause induction, with an obligate diapause or diapause free development as the predominant phenotypes. Thus, *C. fumiferana* is an interesting model system for examining the genetic architecture and regulatory mechanisms of diapause induction and preparation.

Here, we investigate the genetic mechanisms underlying variation in diapause induction in a diapausing and non-diapausing strain of *C. fumiferana*. We compare genome assemblies between the two strains, to investigate whether chromosomal rearrangements play a role. In addition, we investigate the molecular mechanisms underlying the diapause preparation process. We ask at what developmental time point gene expression patterns diverge between diapause- and non-diapause-destined larvae, and what specific gene functions are different between the strains. The availability of a non-diapause colony and genomic tools provides an ideal opportunity to examine the molecular basis of variation diapause induction in *C. fumiferana*. Recently, a chromosome-level assembly of the diapause strain of the spruce budworm was published [26]. Here, we present a chromosome-level genome assembly of a non-diapause strain of *C. fumiferana*, as well as a gene expression comparison with fine temporal resolution between the diapause and the non-diapause strain. Although we do not see evidence of major chromosomal inversions or significant SNP pileups, we did find extensive changes in gene expression between the diapause and non-diapause strain starting midway through the first larval instar. This work sets the stage for future investigation into the *C. fumiferana* diapause phenotype.

## Materials and methods

### Study organisms and tissue sampling

#### Non-diapause laboratory colonies.

Non-diapause *C. fumiferana* were sampled from two strains for this work. The original Harvey [23] strain was established in 1961 from a wild-type (=diapausing) colony of C. fumiferana established from a natural population in Ontario. During the first ~30 years (until the 1990s, exact data unknown), the diapause stock was regularly infused with wild-caught individuals from several populations in Ontario. The line was reared continuously for 60 years at the Insect Production and Quarantine Laboratories, following standard rearing protocols ([27], with one modification: egg masses were put directly on to the McMorran diet for continuous development [28]. This original non-diapause strain, along with a wild-type (=diapausing) laboratory colony (Glfc:IPQL:Cfum; [29]) were used for the comparative RNA-seq analyses described below. The expression of either phenotype was nearly complete (>95%). A second non-diapause strain was again selected from the diapausing laboratory strain using similar selection protocols [23] after the loss of the original non-diapause strain. Individuals from this second strain were collected for whole genome sequencing, Omni-C, and long read transcriptomics. Unfortunately, laboratory disruptions during the COVID pandemic resulted in the loss of the second non-diapause strain and work is underway to re-select a third non-diapause strain from the same diapause strain initially used to develop the previous line.

### Whole genome sequencing

#### DNA extraction for genome sequencing.

High molecular weight DNA was extracted from a 3–4-day old male *C. fumiferana* pupae obtained from the Insect Production and Quarantine Laboratories (Great Lakes Forestry Centre, Sault Ste. Marie, ON, Canada). Pupae were dissected on ice to remove the gut tissue prior to processing with the MagAttract HMW DNA kit (Qiagen, 67563) following the manufacturer’s “Fresh or Frozen Tissue” protocol, where we replaced all vortexing steps with gentle inversions to minimize DNA fragmentation. DNA was quantified with the Qubit dsDNA BR assay on a Qubit 2 fluorometer (Thermo Fisher), then evaluated for purity with a NanoDrop 2000 spectrophotometer (Thermo Fisher) with A260/A280 and A260/A230 ratios above 1.8 as thresholds.

#### Omni-C library preparation.

A single 3–4 day old pupae was used for Omni-C library preparation using the Dovetail (R) Omni-C ® kit (Cantata Bio, Scotts Valley, CA, USA) following manufacturer’s instructions (Omni-C protocol, non-mammalian samples v1.0, Insects & marine invertebrates). The library was sequenced at the Centre d’Expertise et de Services Génome Québec (Montreal, QC, Canada) on an Illumina Novaseq shared S4 lane.

#### Oxford Nanopore sequencing.

Three Oxford Nanopore Technologies (ONT) MinION sequencing runs were pooled to 18.6 gigabases, about 25X coverage assuming a genome size of 600 Mb [26]. For each run, 1 µg of pure genomic DNA was processed with the Ligation Sequencing Kit (SQK-LSK109 or SQK-LSK110). The first run was done on a R9.4.1 flow cell (FLO-MIN106). The second run, also on a R9.4.1 flow cell, was run as previously described, but with DNA treated by the Short Read Eliminator XS size-selection kit (Pacific Biosciences). The third run was as the second one but with a R10.4.1 flow cell (FLO-MIN110). These three sequencing runs yielded 6.5 Gb, 8.5 Gb and 3.1 Gb respectively.

#### Mi-Seq short-read sequencing.

In addition, a whole genome shotgun library was generated with ~500 ng total genomic DNA using the NEBNext Ultra II kit (New England Biolabs). Prior to library preparation, the DNA was sheared to an average size of 600 bp using a Covaris M220 (Covaris). Sequencing was done on a MiSeq apparatus using a 600 cycle v3 kit (2 x 300) at the Plateforme d’Analyse Génomique (Institut de Biologie Intégrative et des Systèmes, Université Laval, Quebec, Canada), leading to 31.6 million read pairs (19.1 Gb in total).

### Genome assembly

#### Draft hybrid assembly.

Both ONT long-read sequencing and MiSeq short-read sequencing datasets were assembled in a long-read-first hybrid assembly approach. Briefly, ONT reads were quality-filtered with NanoFilt v2.6.0 [30] to select only reads above a mean Phred score of 10 and a minimum length of 4000 bp. Likewise, Illumina MiSeq reads were processed with TRIMMOMATIC v0.39 [31] to only select reads above Q20 over at least 36 bp. Filtered ONT reads were assembled de novo with CANU v1.9 [32] using default parameters and an estimated genome size of 600 Mb. The final contigs draft from CANU was polished by aligning the Illumina MiSeq reads using Pilon v1.23 [33]. This step was repeated three times, i.e., until no more draft bases were corrected by Pilon. A final scaffolding step was run with LongStitch v1.0.2 [34] to cut and re-scaffold the draft assembly at potentially misassembled regions.

#### Omni-C scaffolding.

An in-house pipeline was developed to pre-process Omni-C reads, remove internal adapters, and identify true Omni-C contacts usable for bridging contigs. First, Omni-C reads were trimmed with TRIMMOMATIC v0.39 [31] to select only reads above Q30 over a minimum 50 bp length. Then, CutAdapt v3.2 [35] was used to remove the internal adapter bridging the crosslinked Omni-C fragments. The adapter sequence itself was not provided publicly by Dovetail Genomics but was nevertheless identified by analyzing the k-mer frequency among a subset of merged Omni-C read pairs. A 30-mer internal palindrome was found in the top three 31-mers of this subset.

The cleaned Omni-C reads were then aligned to the hybrid draft assembly with Bowtie2 using the preset (--very-sensitive-local) to prevent the loss of Omni-C mappings due to small mismatches and gaps). The resulting BAM files were processed with HiCExplorer v3 [36] to build a Hi-C contact matrix devoid of problematic read pairs (one mate not unique, one mate low quality, one mate unmapped, duplicated pairs, self-ligation, etc.). Given that Omni-C uses a non-specific DNase instead of a restriction endonuclease (“Omni-C 0.1 documentation,” n.d.), the cut sites and dangling sites were set to “NNNN”. Reads used in the final contact matrix were output to BAM format, then used for scaffolding the Canu-Pilon-LongStitch draft with SALSA2 [37], with the genome size (--exp) set at 580 Mb and the cutting enzyme (--enzyme) set at “DNASE”. This procedure was repeated three times, for which contigs below 50 kb, 100 kb and 120 kb were filtered out of the draft at each iteration.

#### Final scaffolding and gap filling steps.

The Omni-C scaffolded draft was further scaffolded by use of the wild-type *C. fumiferana* genome sequence [26] with RagTag version 2.1.0 [38]. Briefly, RagTag corrects probable misassemblies by comparing the draft to a reference, scaffolds the draft over the reference, uses Hi-C information to resolve conflicts and patches gaps. After this step, a final Omni-C scaffolding step occurred as described in the paragraph above, this time without a contig length cutoff applied to the input draft.

### Genome comparison

To test whether major chromosome rearrangements had taken place between the diapause strain (Genbank reference: GCA_025370935.1 [26]) and the non-diapausing strain, we used *Satsuma* [39] to align the respective genomes. The resulting alignments were visually inspected for major rearrangements. The same analysis was also completed using *mummer* [40].

In addition, we called nucleotide variants between the two genomes and looked for elevated SNP occurrences in a sliding window analysis (20k wide, with 10k sliding steps, custom script), where the number of variants were counted in each window. We then ranked windows according to variant frequency, and widows with a high frequency of variants that did not appear to be associated with repetitive elements were inspected further.

### Transcriptomics

#### Short-read transcriptomics.

For gene annotation and gene expression comparison between the two diapause phenotypes, we used comparative transcriptomic data from Béliveau [26] that sampled early life stages (i.e., eggs, 1st, and 2nd instar larvae) from a diapausing *C. fumiferana* strain [29] and the original non-diapause strain [23]. In short, both colonies were reared under standard conditions (16h:8h Light:Dark; 22°C; 60–70% relative humidity) using standard protocols [27]. Under these conditions, eggs generally hatch within 8 days, and molt into 2^nd^ instar after another 5–7 days. They extracted RNA from a pool of 10–60 eggs per time point (sampled 1, 3 and 5 days post-oviposition), a pool of 10–60 1st instar larvae per time point (sampled 1, 3, and 5 days post-hatch) and a pool of 10–60 2nd instar larvae per time point (sampled 1, 3, and 5 days post-molt). Later life stages were also sampled from both strains, including 6th instar larval heads (day 3 post-molt, pool of 20 individuals), two male and two female 5th instars immediately prior to molting, and two males and two female 6th instars (sampled day 2 post-molt). From adult moths, data was reported for male and female antennae, brain, and reproductive tract, as well as the female pheromone gland.

#### Long read transcriptomics.

The whole transcriptome of a 6th instar non-diapause male larva was extracted with the TRIzol method [41] using Thermo Fisher’s TRIzol Reagent. Cell tissues were flash-frozen in liquid nitrogen and ground using a mortar and pestle; afterwards, the manufacturer’s recommended protocol was used. Total RNA was quantified with the Qubit RNA HS kit (Thermo Fisher), and purity was assessed with a NanoDrop 2000 spectrophotometer (Thermo Fisher). RNA was considered pure if A60/A280 and A260/A230 absorbance ratios were above 1.8. Total RNA was then sequenced natively (i.e., without the need for reverse transcription of ribosomal RNA depletion) with Oxford’s Nanopore’s direct RNA sequencing procedure (SQK-RNA004) on a R9.4.1 flow cell (FLO-MIN106). Indeed, direct RNA sequencing adapters bind to poly-A tails in 3’, thereby selectively binding to eukaryotic messenger RNA (SQK-RNA004).

### Genome annotation

Genome completeness was verified using BUSCO [42]. To annotate the genome, we used the MAKER annotation software [43]. In the first round, we used a spruce budworm transcriptome, repeat library and known proteins to inform gene predictions. To create a repeat library, we used repeatmodeler to identify repeats in the genome and create a repeat library. For the transcriptome, we used the same RNA-seq data as reported in Béliveau et al. [26]. Long RNA-seq reads were mapped using minimap2 (settings: -ax -k14 splice). All RNA data was assembled using Trinity using the --long_reads_bam setting to account for long reads in the data [39]. Lastly, we used the protein library from the diapause strain of the spruce budworm as additional input for the first rounds of gene predictions. In the second and third rounds of MAKER iteration, we used both augustus and SNAP training software to improve gene annotations [44,45]. For functional annotations of our gene model we used InterProScan to scan for homology against the uniprot protein database [46–48].

### Differential gene expression

To compare gene expression between the diapausing and non-diapause strains, we used the mRNA-seq data from eggs, L1 and L2 instar larvae as described previously. We investigated which genes were consistently differentially expressed between the diapausing and the non-diapause strain. mRNA reads were aligned to the non-diapause genome, and reads within gene annotations were counted using *featureCounts* from the R package Rsubread [49]. We then used the software *Short Time-series Expression Minor* (*STEM*, [50]) to analyze gene expression. We used *STEM* to cluster gene expression patterns in 20 different temporal expression profiles, following a log2 normalization. To calculate which profiles have significantly more genes assigned then expected, a permutation test was performed: original time point values are randomly permuted, expression values are renormalized, and genes were assigned to most closely matching profiles, and repeated 50 times, to determine the expected number of genes for each profile. Significance was determined after a Bonferroni correction for multiple hypothesis testing and was based on the total number of genes assigned and the expected number of genes assigned. We then determined GO term enrichment for each of the significant profiles (included in *STEM*, based on a Fisher’s exact test). We used *Revigo* [51] to cluster and summarize GO terms to find the representative GO terms.

Next, we compared temporal expression profiles across the different strains. For each non-diapause profile, we determined the number of genes assigned to a different profile in the diapause strain–the intersection of two profiles. Significance of the number of genes in the intersection was computed using the hypergeometric distribution based on the number of total genes assigned to each of the two profiles, and the total number of genes in the experiment. Thus, we identified which gene groups showed different temporal expression patterns, and which GO terms were enriched in those groups. We used *Revigo* [51] to cluster and summarize GO terms to find the representative GO terms.

## Results

### Genome assembly and annotation

We extracted high molecular weight DNA from a non-diapausing adult male *C. fumiferana*, and used both long-read and short-read sequencing for assembling the genome. The genome assembly resulted in 30 full-length scaffolded sequences larger than 6 Mbp, representing a chromosome level assembly. 769 smaller scaffolds were also assembled, with a size ranging from 200 base pairs to ~450 Kbp. BUSCO analysis indicated a 98.5% completeness score (complete + fragmented), of which 3.4% was duplicated. 42.6% of the genome was soft-masked due to the presence of repetitive sequence (Table 1).

**Table 1:** Comparison statistics for the diapause and the non-diapause genome.

	diapause	non-diapause
N50	20.6 Mbp	18.5 Mbp
Assembly size	575.3 Mbp	572.3 Mbp
Scaffolds (>6 Mbp)	30	30
Mbp captured in scaffolds > 6 Mbp	569.6 Mbp	521.4 Mbp
Scaffolds	334	799
Mbp captured in scaffolds < 6 Mbp	5.7 Mbp	50.9 Mbp
Busco score (complete + fragmented)	97.7%	98.50%
Busco score duplicated	3.9%	3.40%

We then used both long- and short-read transcriptomics for gene annotations. The MAKER annotation pipeline of the non-diapause genome resulted in 21,609 gene annotations with 91% of the genes showing an AED-score of 0.5 or better.

### Genome comparisons of the diapause and non-diapause strain

**Fig 2:**
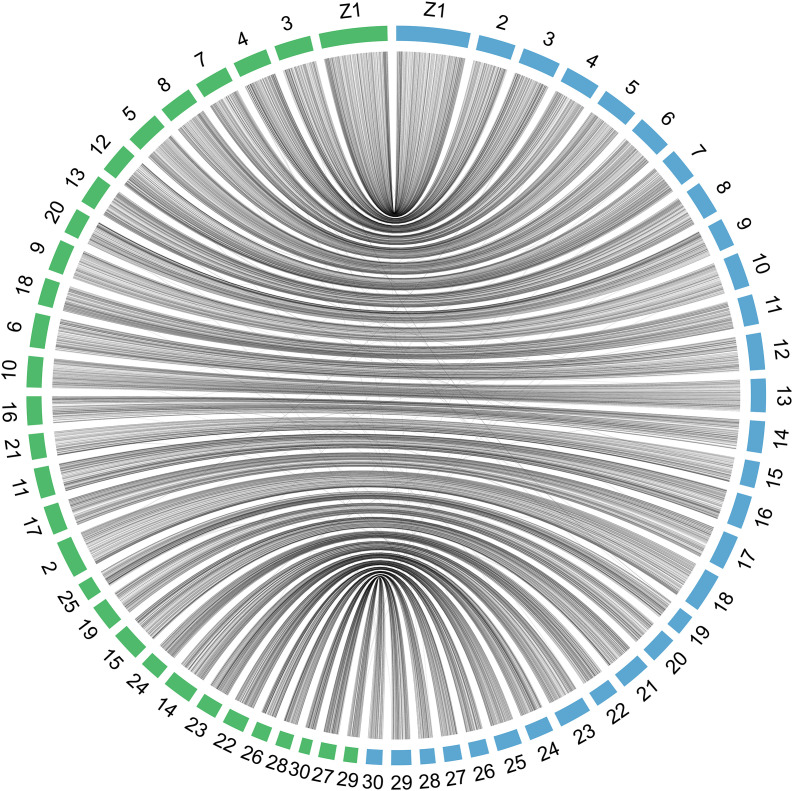
Chromosomal alignment between the diapausing (blue) and non-diapause strain (green). Alignment of two genomes reveals high levels of similarity between genomes. There is no evidence of major chromosomal rearrangements between the two strains.

We performed whole genome alignments using the genomes of the diapausing and the non-diapause strain using both *Satsuma* [39] and *mummer* [40]*.* Genome alignment did not show any evidence of major chromosome rearrangements between the diapausing and non-diapausing strain (Figs 2, S1 in S1 File). We then compared nucleotide variation between the genomes of the two strains to identify concentrated regions of genetic divergence between the strains. Although elevated levels of SNP variation were found on chromosomes 10 and 30, these regions also contained elevated levels of repetitive sequences or had missing nucleotides (Ns) nearby. Both features could artificially increase the number of SNPs due to assembly artifacts.

### Differential gene expression

**Fig 3:**
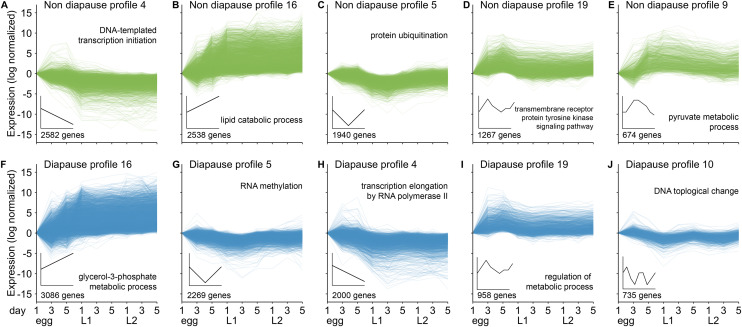
Temporal gene expression profiles for the diapause and non-diapause strain. Spruce budworm larvae were sampled over nine days, from three different life stages: egg, L1 and L2 for each strain; non-diapause (A-E) and diapause (F-J). Similar temporal gene expression profiles were clustered together (black inserts display general shape of expression profile). For each strain, five expression profiles had significantly more genes assigned than expected based on a permutation test (test data shown in Table 2). The number of genes and the top enriched GO term for each profile are shown.

To identify where the gene expression profiles between the diapausing and the non-diapausing line diverge, we used a high-resolution time-series with 9 time-points covering development from egg to early second instar when diapause is initiated. This design heavily emphasizes the time-scale resolution at the cost of replicates per time-point, to allow us to more precisely pinpoint the timing of diapause initiation. As each timepoint represented a pool of a minimum of 10 individuals, we do believe this design allowed for a broad overview of major transcriptomic events in diapause development. We used STEM to identify 20 temporal expression profiles [50]. These profiles are designed by the software to be maximally distinct from one another, such that slight variations in gene expression would still lead to the assignment of a gene to the same profile. We then identified significantly enriched expression profiles for each of the two strains separately. We assigned expression profiles for each gene to 20 different clusters and found that both the diapause and the non-diapause strains had five temporal expression profiles with significantly more genes assigned than expected by a permutation test (Fig 3, Table 2). GO-term enrichment was determined by dividing the number of genes assigned by the number of genes expected. The highest fold-enriched GO term was displayed in Fig 3. For a complete list of GO terms enriched in each profile, see S1 Table.

**Table 2:** Expected number of genes versus assigned number of genes. Expected number of genes is based on a permutation test.

Non-diapause strain profiles	expected	assigned	p-value
Profile 4	658	2582	<0.001
Profile 16	614	2538	<0.001
Profile 5	604	1940	<0.001
Profile 19	670	1267	<0.001
Profile 9	566	674	<0.001
Diapause strain profiles			
Profile 16	628	3086	<0.001
Profile 5	642	2269	<0.001
Profile 4	677	2000	<0.001
Profile 19	682	958	<0.001
Profile 10	574	735	<0.001

**Fig 4:**
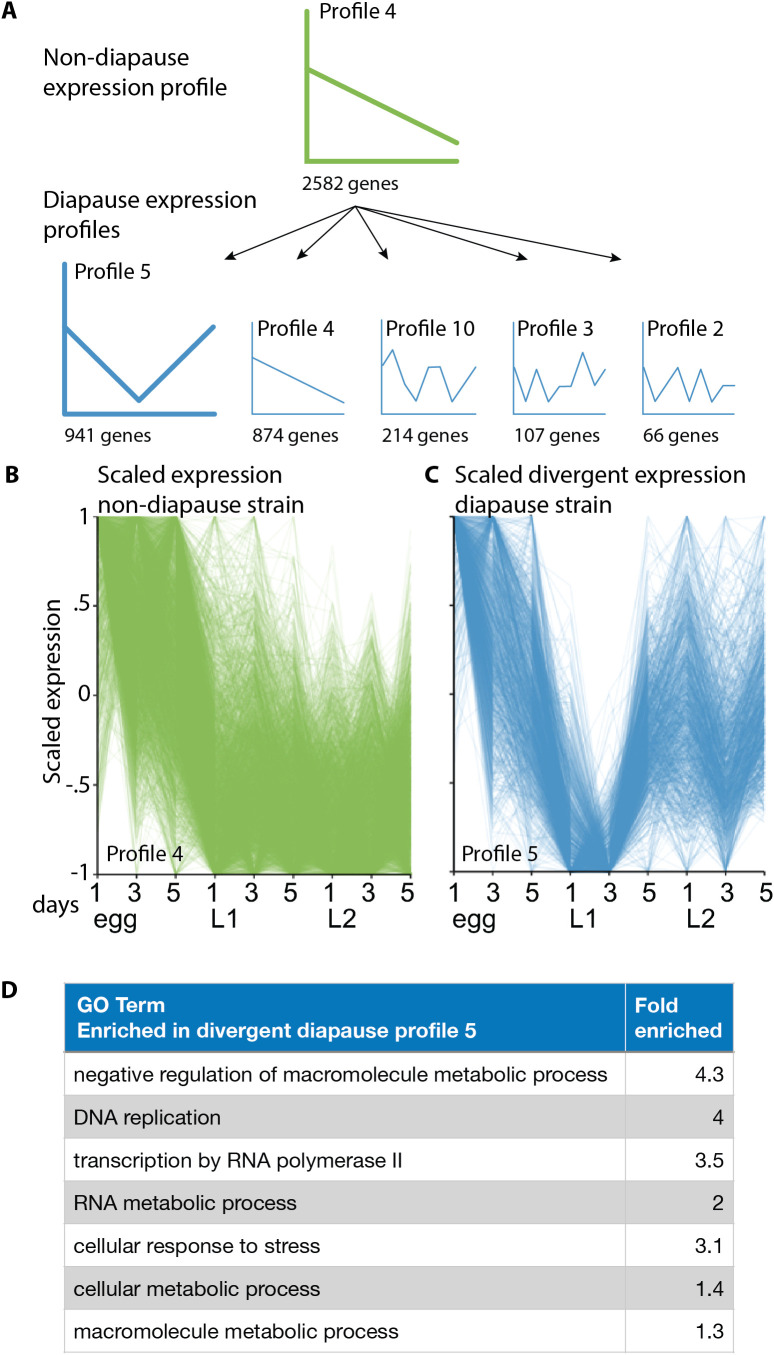
Divergence between the non-diapause strain temporal gene expression profile 4 and the diapause strain. A. Genes assigned to temporal expression profile 4 in the non-diapause strain were assigned to four different profiles in the diapause strain. B. Non-diapause strain profile 4. C. Divergent genes assigned to diapause strain expression profile 5. Data in B and C is scaled from 1 to −1. See SI figure S3 in S1 File for unscaled data. D: GO term enrichment in the divergent gene expression in profile 5. Fold enriched is the total number of genes over the expected number of genes assigned to a GO term.

**Fig 5:**
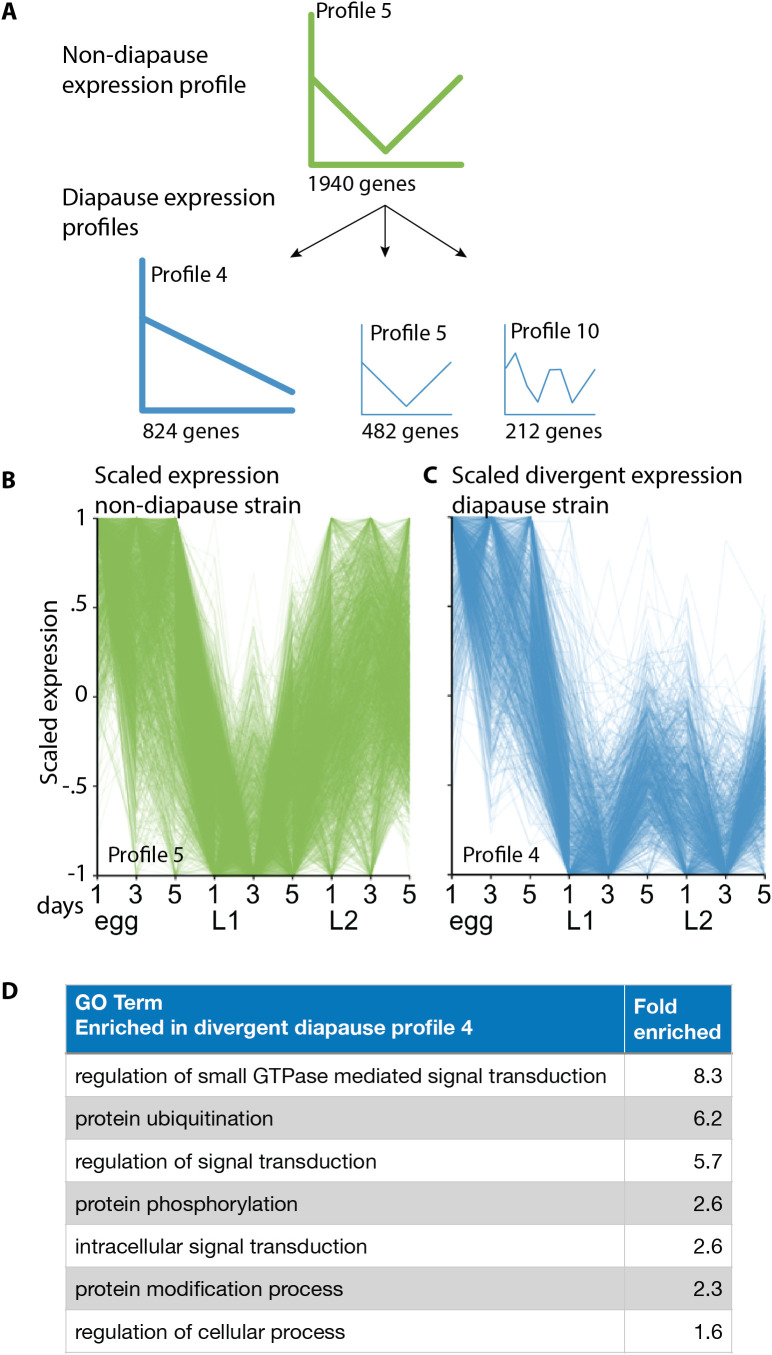
Divergence between the non-diapause strain temporal gene expression profile 5 and the diapause strain. A. Genes assigned to temporal expression profile 5 in the non-diapause strain were assigned to two different profiles in the diapause strain. B. Non-diapause strain profile 5. C. Divergent genes assigned to diapause strain expression profile 4. Data in B and C is scaled from 1 to −1. See SI figure S4 in S1 File for unscaled data D: GO term enrichment in the divergent gene expression profile. Fold enriched is the total number of genes over the expected number of genes assigned to a GO term.

**Fig 6:**
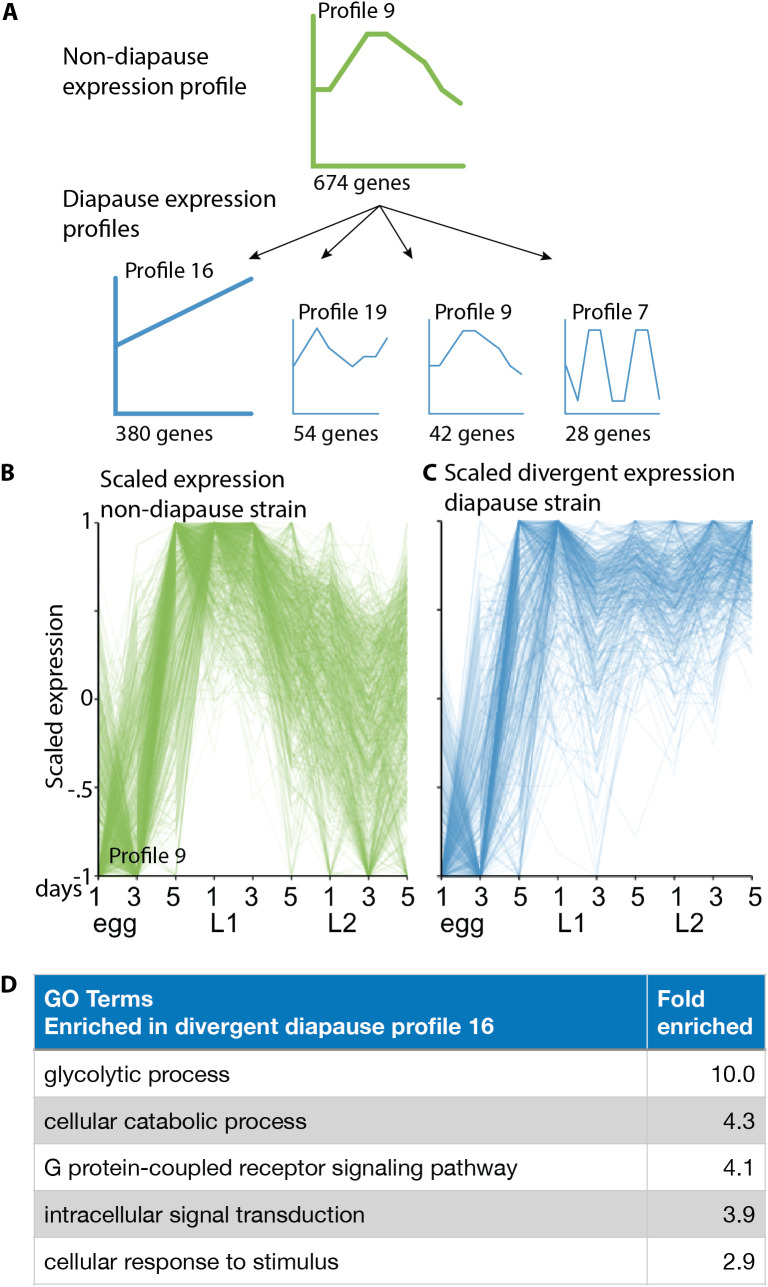
Divergence between the non-diapause strain temporal gene expression profile 9 and the diapause strain. A. Genes assigned to temporal expression profile 9 in the non-diapause strain were assigned to three different profiles in the diapause strain. B. Non-diapause strain profile 9C. Divergent genes assigned to diapause strain expression profile 16. Data in B and C is scaled from 1 to −1. See SI figure S5 in S1 File for unscaled data D: GO term enrichment in the divergent gene expression profile. Fold enriched is the total number of genes over the expected number of genes assigned to a GO term.

We then used the expression profiles of the non-diapause strain as a baseline to determine which genes in the diapause strain showed divergent expression patterns. In other words, we asked STEM to identify which genes that were assigned to one profile in the non-diapause strain were assigned to a *different* profile in the diapause strain. Although our experimental design does not allow for direct differential gene expression comparison due to lack of time point replication, we can identify contrasting expression profiles over time and identify when gene expression diverges between the strains, and which GO terms are enriched in expression differences between the two strains. For three of the five gene expression profiles in the non-diapause strain (Profiles 4, 5, and 9), genes in the diapause strain showed significantly different expression profiles, with more than 10% of genes assigned to different profiles between the diapause and non-diapause strains (Figs 4–6. S2 Fig). We considered these differences to represent genes that could be associated with regulating the diapause phenotype.

In non-diapause profile 4, 2582 genes had expression that continually decreased throughout the sampled timepoints (Fig 4A-B). When contrasted with the non-diapause strain, 874 genes in the diapause strain showed the same expression profile, while 941 genes increased in expression midway through the 1st instar (i.e., Profile 5) (Fig 4C). Diapause profiles 10, 3 and 2 had 214, 107, and 66 genes assigned to them, respectively (Fig 4A). GO term enrichment in the largest differing profile was associated with the negative regulation of developmental processes and DNA replication (Fig 4D for a complete list). Genes assigned to each GO term are reported in S2 Table.

In non-diapause strain profile 5, 1940 genes exhibited expression that decreased during egg and early 1st instar development but increased in expression by the 2nd instar (Fig 5A,B). Of those genes, 482 genes were assigned to the same temporal expression profile (i.e., Profile 5) in the diapausing strain. However, 824 genes showed a different temporal expression profile in the diapausing strain, where expression continued to decline through the 2nd instar (Profile 4, Fig 5C). There were also 212 genes assigned to profile 10, which showed oscillating expression over time (Fig 5A). GO term enrichment in Profile 4 genes was associated with signal transduction and protein ubiquitination (see Fig 5D for a complete list). Genes assigned to each GO term are reported in S3 Table.

In the third non-diapause strain profile (Profile 9), 674 genes exhibited expression that increased in the egg stage and early 1st instar larvae but decreased in expression halfway through the first instar (Fig 6A,B). Only 42 genes were assigned to the same profile in the diapausing strain, while 380 were assigned to profile 16 (continuous increase in expression over time) (Fig 6C). Significantly enriched GO terms included glycolytic process, cellular catabolic processes and cellular signal transduction (see Fig 6D for a complete list). Genes assigned to each GO term are reported in S4 Table.

Because the GO terms for signal transduction (profile 4, decrease in diapause) and intracellular signal transduction (profile 16, increase in diapause) were very similar, and belonged to the same GO term ancestor tree (Supplemental Fig. S3 in S1 File), we decided to further investigate the underlying genes for these terms. The genes associated with signal transduction that decreased in expression in the diapause strain were largely associated with growth and GTPase activity, whereas 40% of all genes associated with signal transduction that increased in expression in the diapause strain were involved with sensory signaling transduction.

## Discussion

Diapause is a complex trait that requires extensive physiological changes in development, physiology, and metabolism. Here, we characterize the genetic mechanisms regulating the non-diapause phenotype in a selected strain of spruce budworm, created through artificial selection on within-population variation of diapause incidence. We provide a chromosome-level genome assembly of the non-diapause strain, allowing direct comparison with a recently published genome of a diapause strain that was used to create the non-diapause strain. Genome comparison of the diapause and the non-diapause strains did not reveal evidence of major chromosomal rearrangements, nor did we see any evidence of a locus of major divergence between the strains. When we investigated the pre-diapause stage leading up to diapause initiation, however, we revealed extensive transcriptional divergence starting approximately halfway through the first instar.

### Genetic divergence

We did not find any evidence of a single locus of large effect that could be responsible for causing a phenotypic shift from diapause to non-diapause development in these two strains. Were the shift in diapause incidence due to a chromosomal inversion, we would have found evidence of this with our whole genome alignments in the form of a break in the synteny between two chromosomes [52]. We did not observe such breaks, and slight divergence from the diagonal is likely due to transposable elements. Further investigation of genomic divergence between the genomes included a sliding window analysis to identify SNP piles-ups, which would appear as an increase in nucleotide variants above the background signal. Artificial selection on a single locus of major effect is expected to result in a single region of high divergence due to a ‘hitchhiking effect’, where nucleotide variants in proximity to the causal SNP are swept along in the selection process due an absence of recombination in that locus (selective sweep, [53]). When we tested for nucleotide divergence between the genomes, we did observe regions of high divergence, but all these regions had high levels of repetitive sequences or had missing nucleotides nearby; both of which can result in an apparent localized accumulation of SNP variants. Although we cannot rule out that such regions are associated with the diapause phenotype, the elevated divergence in these regions is more likely to be an artifact than a real signal. It is also possible there is one causal SNP, but the nucleotide divergence in this region isn’t strong enough to detect through our method of whole genome comparison. A QTL map or GWAS could resolve this issue; as such it is a highly interesting avenue for future research, but outside the scope of the present work.

Although we cannot rule out a single locus of large effect, our current evidence does not point in that direction, which leads us to speculate that the change in diapause in our colonies is polygenic in nature. This would be in direct contrast with numerous other studies pointing towards one or a few genes associated with photoperiod signaling or other external signals [16,17]. However, these results identifying a single locus of large effect could be due to the nature of the analysis: QTL mapping tends to find only a few loci of large effect [10]. Indeed, when a whole genome association study was used to identify genetic variation associated with diapause in the speckled wood butterfly, *Pararge aegeria*, multiple loci of small effect were found in addition to two loci of large effect, indicating the polygenic nature of diapause phenotype [5]. It is of course possible that the artificial selection regime in our system could result in confounding factors that affect our results, for example, a selective sweep as described in the previous paragraph, or reduced recombination due to a limited breeding population. To resolve the genetic architecture of the shift from diapause to non-diapause in spruce budworm, a genome wide association study including many individuals will likely be the best approach.

### Transcriptomic divergence

We investigated gene expression at nine time points comparing the development stages leading up diapause induction in the diapause and non-diapause strain of spruce budworm larvae. For both strains we saw a decrease of expression of genes associated with DNA-templated transcription, and a decrease followed by an increase in expression of genes associated with post translational modification (Fig 3). This change in gene expression activity is likely due to the general development from embryo to second instar larvae. The GO terms differed for profile 16 – increase in gene expression over time – where ‘glycerol-3-phosphate metabolic process’ was enriched in the diapause strain, and ‘lipid catabolic process’ was enriched in the non-diapause strain. This difference is very likely due to the differences in metabolism between the two strains, where diapause-destined spruce budworm do not feed, and rely entirely on glycolytic metabolism during overwintering [19,54]. In addition, diapause-destined spruce budworm start to produce a large amount of glycerol to increase cold-hardiness [55] (Fig 1). This contrasts with the non-diapausing budworm that are starting to feed, and thus are upregulating genes associated with regular developmental metabolism. The other clear difference between the strains was revealed in profile 19, which showed an increase-decrease-increase expression profile. In the diapause strain, this profile was associated with DNA-binding transcription activity, while the non-diapause strain profile was associated with the transmembrane receptor protein tyrosine kinase signaling pathway. There is no clear-cut hypothesis as to why these profiles are enriched for different GO terms, but it is most likely due to the difference in developmental programs between the strains.

Our gene expression results revealed extensive divergence between expression profiles; a large portion of genes assigned to one profile in the non-diapause strain were assigned to different profiles in the diapause strain. Genes that were upregulated in the diapause strain, but not in the non-diapause strain, showed a variety of enriched GO terms. For example, we found a strong enrichment of ‘negative regulation of macromolecule metabolic process,’ which is unsurprising given that downregulation of metabolism is common in diapausing insects. We also saw an upregulation of genes associated with the cellular response to stress, all of which also had the GO term ‘DNA damage response’. This is very likely part of the preparation for diapause, where cold temperatures may lead to an increase in DNA damage and cellular stress [56]. Another expected result was the divergent upregulation of expression of the glycolytic process. As mentioned, diapausing spruce budworm will produce a large amount of glycerol from stored glycogen to increase cold tolerance. Intriguingly, SNP variation in glycerol-3-phosphate dehydrogenase [NAD(+)] was identified in a linkage block that defined genomic differences among eastern and central eastern spruce budworm populations [57]. Given the role that this enzyme plays in glycerol metabolism [58], variation in the induction of cold tolerance invoked by the diapause program may exist among spruce budworm populations.

We were surprised to find a divergent increase in expression of genes associated with DNA replication and transcription in the diapause strain (Fig 4D). Usually, diapause is described as a state of transcriptional shutdown. A meta-analysis of 11 different transcriptomic studies comparing diapausing and non-diapausing insects found consistent downregulation of genes associated with transcription and cell cycle [59], whereas we found an upregulation of these genes (e.g., GO terms ‘DNA replication’ and ‘transcription by RNA polymerase II’). It is possible this discrepancy is due to the difference in sampling times, as our time series encompasses the pre-diapause and early diapause phase, and the meta-analysis focuses on insects already in diapause. This point is further emphasized in a study on the mosquito *Aedes albopictus* [60] which also showed an increase in expression of cell cycle associated genes in the early pre-diapause stage. It could be that we are seeing something similar, that in cell cycling and transcription is increased to prepare for the oncoming period of arrested development during early pre-diapause.

Finally, we observed an upregulation of genes in the diapause strain of spruce budworm associated with environmental cue processing, and more specifically, photoreception. Anecdotally, we have observed that increased light intensity affects the diapause program in our spruce budworm colonies (Roe, unpublished). Interestingly, genetic variation in an ultraviolet-sensitive opsin was also included within the linkage block separating the eastern and central populations of the eastern spruce budworm [57]. These results could be related to the fact that photoperiod is a very common diapause signal. Gene expression and genetic mapping studies regularly point towards circadian clock genes as major factors in diapause evolution [5,8,15,61]. Several other studies show a tight link between opsin expression and circadian clock genes [62–64]. In our study we did not detect an enrichment of circadian clock genes in our divergent expression analysis. However, given the tight link between opsins and circadian clock genes, there is a possible involvement of the circadian rhythm along with other light sensing mechanisms to alter diapause induction.

Surprisingly, the divergence between diapause and non-diapause strain gene expression profiles did not occur until halfway through the first instar. Our findings hint that the diapause-inducing signal could be established earlier, in the embryo or in early first instar larvae. This is reflected in the behavioral phenotype expressed during this period of development, where first instar larvae destined for diapause seek an overwintering site, spin a hibernaculum and clear their gut contents in preparation for diapause and the associated stress of winter [18]. In most species, diapause is a facultative trait, where the signal to induce diapause comes from external conditions such as day length or temperature. During this sensitive period, the external signal is perceived and translated into an internal signal that initiates the diapause program [3].

In the case of the eastern spruce budworm, the second instar diapause is usually obligatory, where larvae will enter diapause regardless of external conditions. However, some variation exists in wild populations, where a small number of larvae will not enter diapause [23]. Although the adaptive value is unclear, it is possible there is some form of bet-hedging, where the non-diapausing larvae can enter diapause at a later stage with increased resources or use this flexibility to avoid phenological mismatch in marginal climates. In addition, the incidence of the non-diapause phenotype can be increased with early exposure to 24 hour light conditions (Cusson, Roe, unpublished), which indicates that diapause induction is facultative for at least part of the natural population. We believe that all of this can be explained by the presence of one type of diapause induction signal, and it can rapidly evolve from ever-present, to facultative, to absent altogether. This mechanism of evolution of induction signaling is common in nature, for example in flesh fly species (Diptera: Sarcophagidae) [65], where some tropical species of flesh fly do not induce diapause, but other species do, although the inducing cue may be different (i.e., Temperature in more tropical species, versus photoperiod in temperate species) [66]. Diapause induction varies in other species, such as *Culex pipiens* [67], and *Daphnia pulex* [68]. Taken all this together, the emerging pattern appears to be that the signal to initiate diapause can evolve readily. The spruce budworm diapause and diapause free strains present a unique opportunity to study this mechanism of evolution on within population variation. Future investigation into the molecular mechanisms of diapausing and non-diapausing strains of spruce budworm will help us understand the minimal changes necessary to alter the diapause phenotype in this widespread forest species.

## Supporting information

S1 File Supporting images S1–S6. (DOCX)

S1 Table GO terms enriched in common expression profiles in the diapause and non-diapause strain. (XLSX)

S2 Table GO terms enriched in diapause profile 5, divergent from non-diapause profile. This gene set contains genes that decrease then increase in expression in diapause, and continue to decrease in expression non-diapause. Rows: indivdual genes, Columns: enriched GO terms. Bold terms are terms presented in Fig 4. Color groups indicate overlap in gene sets.
(XLSX)

S3 Table GO terms enriched in diapause profile 4, divergent from non-diapause profile. This gene set contains genes that continue to decrease in expression in diapause, and decrease then increase in expression non-diapause. Rows: indivdual genes, Columns: enriched GO terms. Bold terms are terms presented in main Fig 4. Color groups indicate overlap in gene sets.
(XLSX)

S4 Table GO terms enriched in diapause profile 16, divergent from non-diapause profile. This gene set contains genes that increase in expression in diapause, and increase then decrease in expression non-diapause. Rows: indivdual genes, Columns: enriched GO terms. Bold terms are terms presented in main Fig 4. Color groups indicate overlap in gene sets.
(XLSX)
