# Supplementary material for: Major changes in gene expression between strains of diapausing and non-diapause spruce budworm
Source: PLoS One. 2026 Aug 18;21(8):e0349357. doi: 10.1371/journal.pone.0349357 (PMC13484964; doi:10.1371/journal.pone.0349357)
Supplement: Supplementary file 1 [file pone.0349357.s001.docx]

## SI figures

 **Supplemental figure 1.**

**Chromosome alignment of the diapause (x-axis) and non-diapause (y-axis) genome.**

No major chromosome inversions were detected.

**Supplemental figure 2.**

**Short Time-series Expression Miner (STEM) output.**

Non-diapause expression profiles are shown on the left of the yellow bar. On the right of the yellow bar the *same* geneset is shown, but with the diapause expression profiles assignments. Black lines show expression profiles, red lines are expression patterns of individual genes.

**Supplemental figure 3: Divergence between the non-diapause strain temporal gene expression profile 4 and the diapause strain. Log normalized data, unscaled**

A. Genes assigned to temporal expression profile 4 in the non-diapause strain were assigned to four different profiles in the diapause strain. B. Non-diapause strain profile 4. C. Divergent genes assigned to diapause strain expression profile 5. D: GO term enrichment in the divergent gene expression in profile 5. Fold enriched is the total number of genes over the expected number of genes assigned to a GO term.

**Supplemental figure 4: Divergence between the non-diapause strain temporal gene expression profile 5 and the diapause strain. Log normalized data, unscaled**

A. Genes assigned to temporal expression profile 5 in the non-diapause strain were assigned to two different profiles in the diapause strain. B. Non-diapause strain profile 5. C. Divergent genes assigned to diapause strain expression profile 4. D: GO term enrichment in the divergent gene expression profile. Fold enriched is the total number of genes over the expected number of genes assigned to a GO term.

**Supplemental figure 5: Divergence between the non-diapause strain temporal gene expression profile 9 and the diapause strain. Log normalized data, unscaled**

A. Genes assigned to temporal expression profile 9 in the non-diapause strain were assigned to three different profiles in the diapause strain. B. Non-diapause strain profile 9. C. Divergent genes assigned to diapause strain expression profile 16. D: GO term enrichment in the divergent gene expression profile. Fold enriched is the total number of genes over the expected number of genes assigned to a GO term.

**Supplemental figure 6**

GO-terms for regulation of signal transduction (enriched in divergent profile 4, fig 5) and intracellular signal transduction (enriched in divergent profile 16, fig 4) were very similar, and belonged to the same GO-term ancestor tree.
