# Supplementary material for: Major changes in gene expression between strains of diapausing and non-diapause spruce budworm
Source: PLoS One. 2026 Aug 18;21(8):e0349357. doi: 10.1371/journal.pone.0349357 (PMC13484964; doi:10.1371/journal.pone.0349357)
Supplement: Supplementary file 2 [file pone.0349357.s007.docx]

Based on the review reports, authors should consider the following points before revision submission.

Specificity of terminology is strongly suggested for clarity in different sections of the manuscript.
Nomenclature was changed throughout to indicate that samples belonged to either a diapausing or a non-diapausing strain.

Develop a strong background leading to stepwise methodology and especially the critical discussion to strengthen your findings with the previous findings.
We have made substantial changes to our introduction. We discussed the three stages of diapause, and focus our introduction more on the pre-diapause stage; which includes diapause initiation and preparation, the processes we investigate in our study.

conclusion should be to the point and based on the findings of the study.

We have softened our conclusions to:

Our findings hint that the diapause-inducing signal could be established earlier, in the embryo or early first instar.

Please use the space provided to explain your answers to the questions above. You may also include additional comments for the author, including concerns about dual publication, research ethics, or publication ethics. (Please upload your review as an attachment if it exceeds 20,000 characters)

Reviewer #1: I believe there are a few questions and points must be addressed prior to publication.
1) The introduction would benefit from more background on insect diapause.

As mentioned also in the comments to the editor, we have added text to the introduction on the pre-diapause stage.

2) The material and methods section did not clearly describe how the non-diapause strains were obtained, what percentage of the non-diapause larvae were induced, and what the variations between generations were. These points are essential in being able to compare the gene expression between the non-diapause and diapause strains.

We have added additional text in the materials and methods on the creation of the strains: In each strain, the expression of either the diapause or non-diapause phenotype was near complete (>95%).

Also, we would like to emphasize that each RNA-seq sample consisted of 10-60 individuals. Thus, the chance of accidentally sampling a non-diapause individual as a diapausing individual or vice versa is low, and if it happened, the signal would likely be drowned out by the other, phenotype conforming samples.

3) Does someone's lab maintain these non-diapausing strains?

We included the following text:

A non-diapause strains of *C. fumiferana* were continuously maintained at the Insect Production and Quarantine Laboratories at the Great Lakes Forestry Centre. Unfortunately, laboratory disruptions during the COVID pandemic resulted in the loss of the second non-diapause strain, and work is underway to re-select a third non-diapause strain from the same diapause strain initially used to develop the previous line.

We would like to mention to the reviewers only that while the original line on which this data was collected unfortunately no longer exists, we still think there is value and interest in analyzing this line. Additional research plans are currently being made to investigate the upcoming lines in more detail.

4) Discussion section should link current research to previous research.

We have added the following text to the discussion (last paragraph).

*We believe that all of this can be explained by the presence of one type of diapause induction signal, and it can rapidly evolve from ever-present, to facultative, to absent altogether. This mechanism of evolution of induction signaling is common in nature, for example in flesh fly species (Diptera: Sarcophagidae) (Denlinger and Shukla, 1984), where some tropical species of flesh fly do not induce diapause, but other species do, although the inducing cue may be different (i.e.. Temperature in more tropical species, versus photoperiod in temperate species (Denlinger 1979). Diapause induction varies in other species, such as* Culex pipiens *(Field, 2022), and* Daphnia pulex *(Otake, 2024). Taken all this together, the emerging pattern appears to be that the signal to initiate diapause can evolve readily. The spruce budworm diapause and diapause free strains present a unique opportunity to study this evolution on within population variation. Future investigation into the molecular mechanisms of diapausing and non-diapausing strains of spruce budworm will help us understand the minimal changes necessary to alter the diapause phenotype in this widespread forest species.*

5) There is not enough data to support the conclusion that the signal that induces diapause occurs early in the first instar.

As mentioned also in the comments to the editor, we have softened our conclusions to:

Our findings hint that the diapause-inducing signal could be established earlier, in the embryo or early first instar.

Reviewer #2: This is an interesting paper that utilizes a unique comparison of spruce budworm individuals from lines that are either diapause capable or diapause incapable to identify genetic architecture of diapause and gene expression leading up to diapause. This provides a comparison that contrasts with most published work on diapause gene expression that looks at variation across natural gradients. The authors found that there are no clear major genetic rearrangements responsible for the differences and there are differences observed in gene expression leading up to diapause. It is a novel study and a well written paper. I enjoyed reading it, but I do have some comments regarding the interpretation of results and the terminology used.

We thank the review for their kind comments.

The major comments are:

1) First, I think that the names of the strains are a bit misleading. Diapause is a developmental process, and what is being compared is the genomes and gene expression of individuals in development mostly before they are in diapause. This can lead to potentially misusing the names of the strains with the process, like on L368, when it is said that “...upregulation of genes in diapausing spruce budworm...”, where the expression was not measured in individuals in diapause. This is mostly avoided by referring to them as diapause strain though. I would recommend making this more clear by calling the strains diapause capable or diapause incapable, or something like that.

We agree that this was confusing at times, and we have updated the nomenclature in our paper to specify diapause strain or non-diapause strain.

2) I am also struggling a bit to wrap my head around what question the experimental design is addressing.

We added additional text to the introduction and the discussion that we’re specifically looking at the molecular events during the pre-diapause stage, comparing diapause and non-diapause strains.

Generally, the expression papers that are cited as comparisons here are looking at natural variation in diapause cues that adapt to local environment. I would consider diapause inducing cues to be different from diapause or diapause regulation, they are linked, but not the same. Populations that have differing critical photoperiods would differ when they enter, but once they are in diapause the diapause is likely very similar. So then what is being compared between your strains? It is a little complicated by them being obligate, but still able to respond to extreme photoperiod (L395). Do the individuals selected in the non-diapause strain lack the ability to trigger the induction phase, or is it something else in the cascade leading to diapause? If you are looking at more complicated trait, then it could explain why in L301 says your findings are in contrast to other studies which have loci of large effect when they are looking at photoperiod or other cues.

Yes, the individuals in the non-diapause strain appear to lack the ability to enter diapause. We’d like to point out that it is very difficult to distinguish between a complete lack of ability to enter diapause, or the cue necessary to induce diapause is so extreme that falls outside normal environmental conditions. Essentially, there could be an extreme condition that could trigger diapause induction, but we don’t know what it is.

We believe that the most parsimonious explanation is that the diapause regulatory cascade is still hard-coded in the non-diapause strain, due to the ease of selection, but the trigger to start the induction phase is no longer there. However, we definitely need more research to resolve these questions!

We adjusted the discussion:

We believe that all of this can be explained by the presence of one type of diapause trigger, and it can rapidly evolve from ever-present, to responsive, to absent altogether.

3) I also wonder about the function of individuals in a population that do not enter diapause. I would expect they would have very low fitness as they likely have low survival. Would it be some form of bet hedging? There is often strong selection on critical photoperiod because it is important for survival to enter diapause at the right time, and since there are loci of large effect underlying these traits usually, selection acts on it quickly. This also may explain why you do not find strong evidence of loci of large effect. I do not mean for this comment and the last to sound critical, I find it to be a very interesting part of the system and study, and perhaps would have liked a little bit more of it in the discussion.

Yes! We believe there is some form of bet-hedging going on. For example, spruce budworm have the ability to enter a second, facultative diapause in the 4^th^ instar (Harvey 1961). It is possible that a portion of the population could initially forego diapause at the 2^nd^ instar, choosing instead to initiate feeding via needle mining to gain additional resources for winter. Then that larvae could enter diapause at a second stage later in development, at the 4^th^ instar. That is highly speculative though, as that hypothesis has not been tested. It is valuable to note that variation in the diapause program is observed within the Choristoneura species complex. The two cycle budworm (Choristoneura occidentalis biennis) has two obligate diapause periods – 2^nd^ instar and 4^th^ instar. This means it requires two years to complete development. Phenotypic variation in the diapause program makes Choristoneura such an interesting model system, thus our interest in the underlying genomic architecture and molecular programming.

We have added the following text to the discussion:

Although the adaptive value is unclear, it is possible there is some form of bet-hedging, where the non-diapausing larvae can enter diapause at a later stage with increased resources or use this flexibility to avoid phenological mismatch in marginal climates.

4) I find Figures 4-6 to undermine the methods a bit. I am not questioning the validity of the methods used, but the data shown in these figures rely on having a different expression profile, and in every case, they seem to be very similar expression profiles when shown in panels B and C. I was thinking that maybe making a supplementary figure of an example of several of the discussed transcripts in non-diapause and diapausing strains to show their divergent expression. The differences may look more convincing when not grouped with hundreds of others. This may give the readers some reassurance that they differ. This is just a suggestion though, and I think it is ok without it.

We agree that when looking at so many genes together, the signal gets lost in the noise. We are very hesitant to show plots of individual genes. Because each timepoint only has one replicate, results for individual gene are very underpowered. We only really have power when doing bulk analysis. However, we did find that when we scaled all genes to the same scale, from -1 to 1, this clarified the signal. We have replaced the comparison figures in 4-6 with the scaled versions, and put the original figures in the SI.

Minor comments:

L28: Would this be “...a small subset of individuals do not diapause...”?

Yes, we have made the edit

L72: I see that here you mention that you are looking at diapause induction and not diapause in general, maybe this needs to be clarified throughout.
We have made edits throughout the paper to make this point more clear.

L355: There is no panel F in Figure 4,
We have changed this to 4D

L395: I think that it would be useful to add that they also can be induced to skip diapause when exposed to long photoperiod in the introduction.

We have added a sentence in the introduction

Reviewer #3: This well written manuscript explores the differences between diapausing and non-diapausing spruce budworm in the genome as well as in the transcriptome. This approach to study multiple genetic traits behind the change from obligate to facultative and in the end no diapause within a one population is appealing. Here the authors present a study in which they were able to show that the change in life history decision comes mainly from the transcriptome and not genetic variation. The time points for the gene expression were smartly chosen to get as close to this decision as possible. Overall, I think this is a well-designed experiment with good execution and written up in a concise manuscript. Therefore, as a reviewer I would suggest this manuscript for publication, after the minor comments have been addressed by the authors.

Line 62 & 71: Responsive diapause is more commonly termed facultative diapause and it might be better to talk about facultative diapause that is responsive instead of responsive diapause.’

We have changed responsive diapause to facultative diapause throughout

Line 91 – 116: Please go through the text and put all the species names in italic. Requested edits have been made.
Line 168: If you follow kostal 2006 diapause is first induced and then initiated and as I understand diapause is induced in the first instar and initiated in the early second instar in your species. Could you change from induced to initiated. Requested edits has been made
Did you sample the eggs and instars always at the same time after lights on, as you were looking for changes in circadian clock genes?

The samples were taken during the day but were synchronized to a set of hours after egg laying (with a margin of 6 hours). So no, the samples were not all taken at the same time of day. We also don’t draw conclusions on whether clock genes are differentially expressed, as that would indeed be problematic. Future experiments will take this into account.

Figure 1 or methods: could you describe the timeframe for the insects how long the egg, L1 and L2 stage last. That would make it easier to assess the scope of the transcriptomic changes.

We have added text to the materials and methods:
Under these conditions, eggs generally hatch within 8 days, and molt into 2^nd^ instar after another 5-7 days. They extracted RNA from a pool of 10-60 eggs per time point (sampled 1, 3 and 5 days post-oviposition), a pool of 10-60 1st instar larvae per time point (sampled 1, 3, and 5 days post-hatch) and a pool of 10-60 2nd instar larvae per time point (sampled 1, 3, and 5 days post-molt).
